# Bone Marrow-Derived Mesenchymal Stem Cells Modified with Akt1 Ameliorates Acute Liver GVHD

**DOI:** 10.1186/s12575-019-0112-2

**Published:** 2019-12-16

**Authors:** Lukun Zhou, Shuang Liu, Zhao Wang, Jianfeng Yao, Wenbin Cao, Shulian Chen, Wenjun Xie, Shuqing Feng, Yuanfu Xu, Tao Cheng, Mingzhe Han, Sizhou Feng

**Affiliations:** 1 State Key Laboratory of Experimental Hematology, National Clinical Research Center for Blood Diseases, Institute of Hematology & Blood Diseases Hospital, Chinese Academy of Medical Sciences & Peking Union Medical College, 288 Nanjing Road, Tianjin, China; 2 Guangzhou Medical University, 195 Dongfeng Xi Road, Guangzhou, Guangdong Province China; 3 North China University of Science and Technology Affiliated Hospital, 73, Construction South Road, Lubei District, Tangshan, Hebei Province China

**Keywords:** AKT1, mesenchymal stem cells (MSCs), Liver aGVHD, ConA-induced liver injury, Cell therapy

## Abstract

**Background:**

Liver injury associated with acute graft-versus-host disease (aGVHD) is a frequent and severe complication of hematopoietic stem cell transplantation and remains a major cause of transplant-related mortality. Bone marrow-derived mesenchymal stem cells (BM-MSCs) has been proposed as a potential therapeutic approach for aGVHD. However, the therapeutic effects are not always achieved. In this study, we genetically engineered C57BL/6 mouse BM-MSCs with AKT1 gene and tested whether AKT1-MSCs was superior to control MSCs (Null-MSCs) for cell therapy of liver aGVHD.

**Results:**

*In vitro* apoptosis analyses showed that, under both routine culture condition and high concentration interferon-γ (IFN-γ) (100ng/mL) stimulation condition, AKT1-MSCs had a survival (anti-apoptotic) advantage compared to Null-MSCs. *In vivo* imaging showed that AKT1-MSCs had better homing capacity and longer persistence in injured liver compared to Null-MSCs. Most importantly, AKT1-MSCs demonstrated an enhanced immunomodulatory function by releasing more immunosuppressive cytokines, such as IL-10. Adoptive transfer of AKT1-MSCs mitigated the histopathological abnormalities of concanavalin A(ConA)-induced liver injury along with significantly lowered serum levels of ALT and AST. The attenuation of liver injury correlated with the decrease of TNF-α and IFN-γ both in liver tissue and in the serum.

**Conclusions:**

In summary, BM-MSCs genetically modified with AKT1 has a survival advantage and an enhanced immunomodulatory function both *in vitro* and *in vivo* and thus demonstrates the therapeutic potential for prevention and amelioration of liver GVHD and other immunity-associated liver injuries.

## Background

Acute graft-versus-host disease (aGVHD) develops in a significant number of patients who receive allogeneic hematopoietic stem cell transplantation (allo-HSCT), and liver is one of the most commonly affected organs [1–3]. Liver aGVHD is caused by the generation of alloreactive T cells that migrate to liver and induce liver injury [4]. It is a major cause of complications and death in patients who receive allo-HSCT and thus effective therapeutic strategies are needed [2]. Currently, the standard first-line treatment for aGVHD is immunosuppressive drugs, such as methylprednisolone and cyclosporine A. However, these drugs are often associated with secondary infections and about half of the patients do not respond to these drugs. So far, no effective strategy for steroid-refractory aGVHD has been established and new therapeutic strategies for these patients remain to be developed [5].

MSCs is a very promising treatment for aGVHD due to its immunosuppressive function. Recent studies have shown its effectiveness in many cases of aGVHD, especially in steroid-refractory aGVHD. However, the therapeutic effects are not always achieved [6–10]. Genetically manipulating MSCs *in vitro* has promising potential in improving MSCs' functions to maximize its treatment potential, as shown by several studies [11–15]. AKT1 is a serine/threonine kinase that plays a key role in the modulation of cell proliferation and survival. It is well known for its anti-apoptotic effects against a variety of situations including oxidative and osmotic stress, irradiation and ischemic shock [16]. Recent studies have demonstrated that AKT1 played a pivotal role in regulating MSCs migration and secretion of paracrine cytoprotective factors [17–19]. In addition, Mangi and colleagues reported that AKT1-overexpressed MSCs were more resistant to apoptosis and could better prevent cardiac remodeling and restore the performance of infarcted hearts after transplantation into the ischemic rat heart [20]. Further study revealed that enhanced paracrine action of AKT1-MSCs accounted for MSCs function improvement [18, 19].

The inflammatory microenvironment plays a crucial role in the activation of MSCs. IFN-γ, a potent pro-inflammatory cytokine produced by activated T-cells, NK cells, NKT cells and macrophages, has impacts on many properties of MSCs, such as cell proliferation, differentiation, apoptosis and senescence [21–23]. It is also an inducer of the chemokine secretion and adhesion molecule expression of MSCs, which partially accounts for MSCs immunosuppressive and tissue reparative function [24–26].

In this study, we overexpressed AKT1 in mouse BM-MSCs and evaluated its role in regulating cell viability and paracrine function under IFN-γ-stimulated condition. For *in vivo* study, we used a ConA-induced liver injury model to imitate liver aGVHD as they are similar in terms of the hepatotoxic mechanism, as both are induced by polyclonally activating T cells. We aimed to investigate whether AKT1-MSCs was superior to control MSCs (Null-MSCs) in resistant to apoptosis and amelioration of immune-mediated hepatitis induced by ConA, as well as to ascertain the potential mechanisms of these effects.

## Results

### MSCs Culture and Characterization

**Fig. 1:**
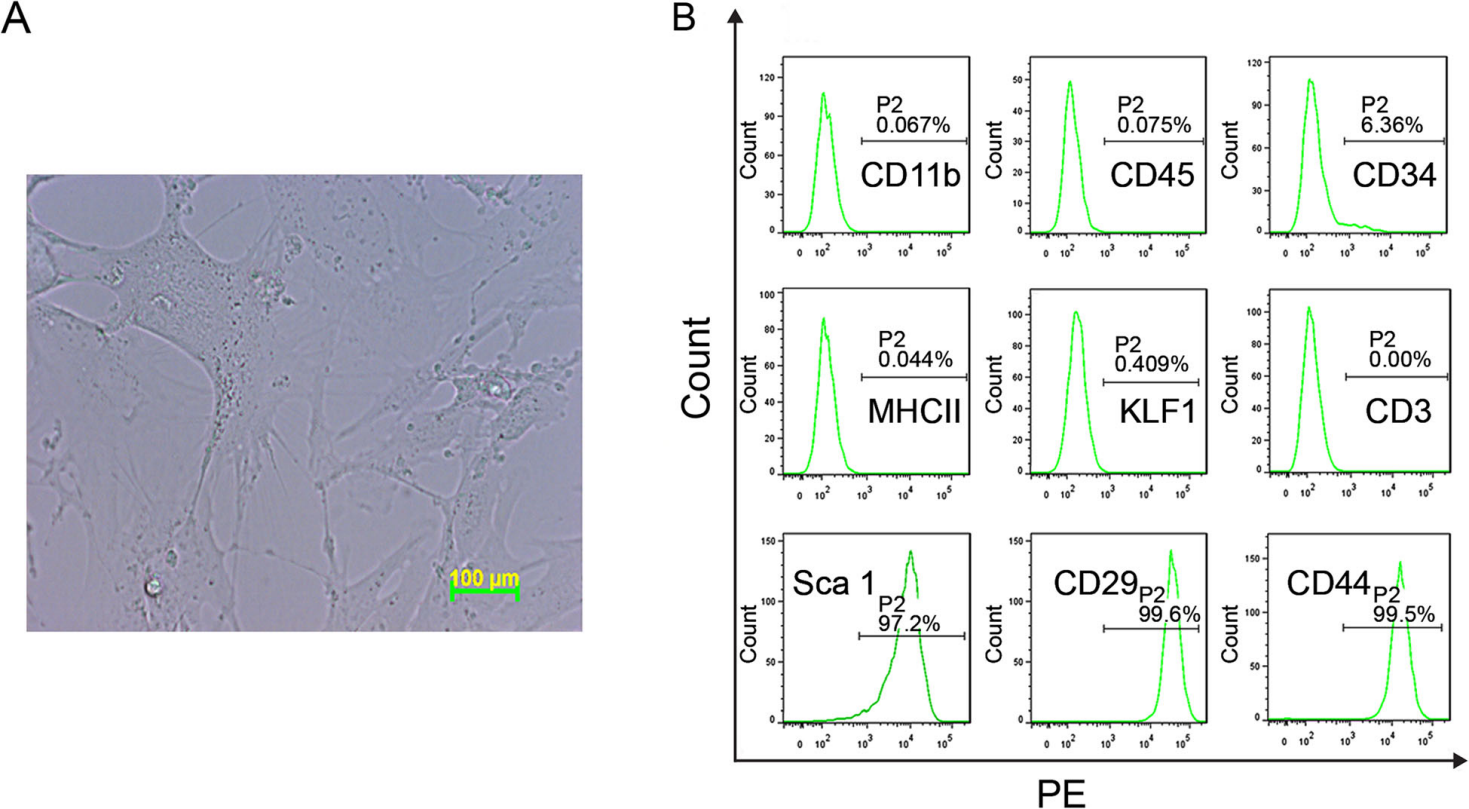
Mesenchymal stem cells culture and characterization. **a** The morphology of the third passage of MSCs in culture. Scale bar represented 100 μm. **b** Phenotypic characterization of the third passage of MSCs. Flow cytometry analysis was performed with PE-conjugated antibodies against MSC markers CD29, CD44, Sca-1; hematopoietic markers CD11b, CD3, CD45, CD34 and MHC II, KLF1. PE-isotype control antibody was used for setting gates

MSCs isolated from C57/B6 mouse bone marrow were obtained from the Cyaen company. These cells could expand for up to 20 passages. We analyzed the third passage of MSCs for cell morphology and cell surface markers. As shown in Fig. 1a, MSCs morphology was similar to fibroblasts which were fusiform or irregular triangle shaped. These cells had ovoid nuclei and 2 to 3 cytoplasmic processes of various lengths. Phenotypic analysis by flow cytometry demonstrated that these cells were positive for MSC markers CD29, CD44, Sca-1 and negative for major histocompatibility complex II (MHC II), kruppel-like factor1 (KLF1) and hematopoietic markers CD11b, CD3, CD45 and CD34 (Fig. 1b).

### MSCs Genetically Modified with AKT1 Gene

**Fig. 2:**
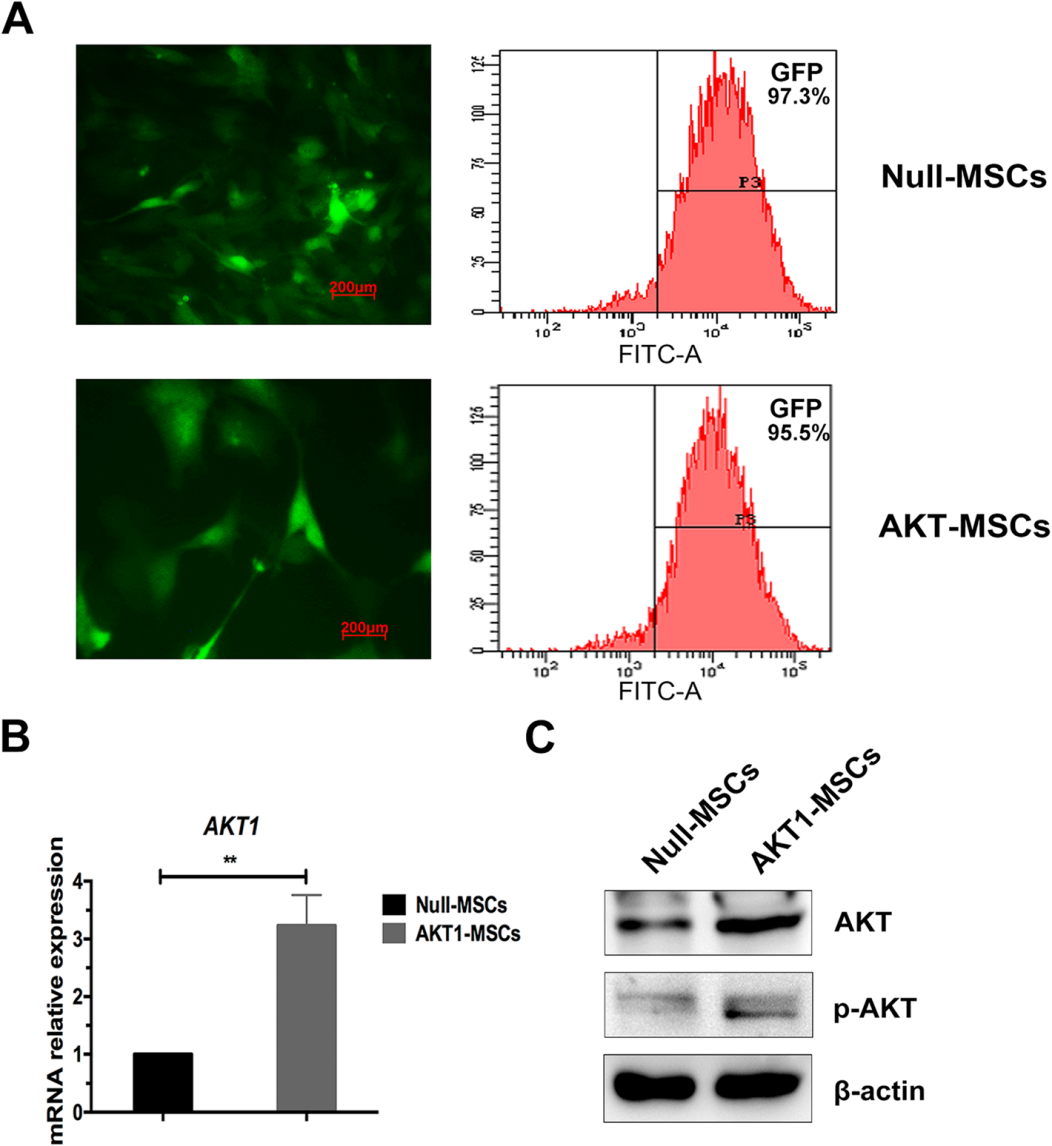
Over-expression of AKT1 in MSCs. **a** At 72h after infection, the transduction efficiency of Null-MSCs and AKT1-MSCs was evaluated by fluorescence microscopy (*left*, Scale bar = 200μm) and flow cytometry (*right*). Non-infected cells were used as the control for setting gates. **b** At 72h after infection, the infected cells were sorted by flow cytometry for GFP+ population. The mRNA expression levels of AKT1 were quantified by qRT-PCR, normalized to those of GAPDH and set to 1 in Null-MSCs group. The data are presented as the means ± SD of three independent experiments. *P < 0.05, **P < 0.01, ***P < 0.001, ****P < 0.0001. C. Representative western blots showing total AKT1 and phosphorylated AKT1 expression in Null-MSCs and AKT1-MSCs

We used retroviruses to transduce MSCs with Null-GFP gene (Null-MSCs) and AKT1-GFP fusion gene (AKT-MSCs), with over 95% efficiency (Fig. 2a). The GFP+ cell population was flow-sorted and the expression of AKT1 was tested by real-time polymerase chain reaction (qRT-PCR) and western blotting. qRT-PCR showed that AKT1 mRNA was about three-fold higher in AKT1-MSCs compared with Null-MSCs (Fig. 2b). Over-expression of total AKT1 and phosphorylated AKT1 was further confirmed by Western blotting as shown in Fig. 2c. These data indicated successful incorporation of exogenous AKT1 gene into MSCs.

### Cytoprotective Effect of AKT1 Over-Expression on MSCs

**Fig. 3:**
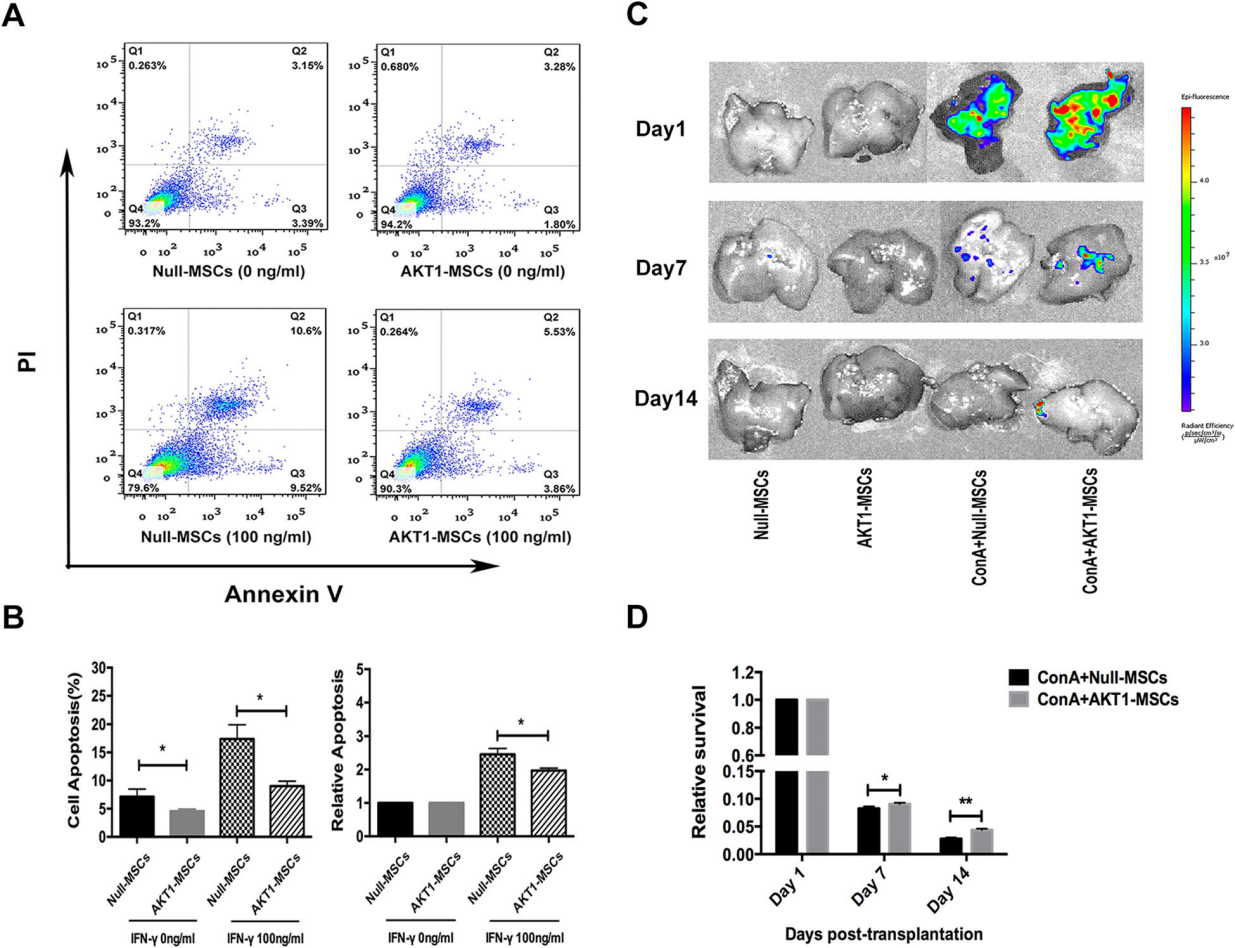
Cytoprotective effect of AKT1 over-expression on MSCs. **a** Representative flow cytometry scatter plots depicting the percentage of apoptotic cells in Null-MSCs and AKT1-MSCs group under routine culture condition and IFN-γ stimulation condition. **b** The percentage of Annexin V-positive cells in each group is indicated in a bar chart (*left*). The ratio of Annexin V-positive cells under IFN-γ stimulation condition relative to untreated controls is indicated in a bar chart (*right*). The data are presented as the means ± SD of three independent experiments. *P < 0.05, **P < 0.01, ***P < 0.001, ****P < 0.0001. **c** Liver fluorescence images on day1, day7 and day14 after transplanting Null-MSCs or AKT1-MSCs into mice with or without Con A administration. **d** Relative quantification analysis of Null-MSCs and AKT-MSCs on day7 and day14 in ConA administration group. Fluorescence signals were quantified by ROI analysis using Living Image software 4.2 and normalized to those of Day1 in each group

To test whether AKT1-engineered MSCs were more resistant to apoptosis than Null-MSCs, we performed apoptosis assessment of MSCs by Annexin V/PI staining. There was a slight decrease in apoptosis in AKT1-MSCs as compared with Null-MSCs under routine culture condition (Fig. 3a, b). We then investigated the survival capacity of MSCs under inflammatory circumstance. Null-MSCs and AKT1-MSCs were stimulated with IFN-γ (100ng/ml) for 24h, and then collected for apoptosis analysis. As shown in Fig. 3a, b, the percentage of apoptotic cells was significantly lower in the AKT1-MSCs group than that in Null-MSCs group after IFN-γ stimulation.

To further assess the cytoprotective effect of AKT1 over-expression on MSCs *in vivo*, we intravenously injected 5 × 10^6^ Null-MSCs or AKT1-MSCs into mice with or without ConA administration, respectively. At day1 post-transplantation, a strong fluorescence signal was emitted from the liver in both Null-MSCs and AKT1-MSCs groups with ConA administration and no signal was detected in mice without ConA administration, suggesting the homing capability of MSCs to the damaged area. The fluorescence intensity was much stronger in AKT1-MSCs group than that in Null-MSCs group, which indicated that AKT1-MSCs have a better homing ability than Null-MSCs. At day 7 and day 14 after transplantation, though the fluorescence signal faded in both groups, it was still stronger in AKT1-MSCs group than that in Null-MSCs group, suggesting AKT1-MSCs survived better than Null-MSCs *in vivo* (Fig. 3c, d).

### AKT1 Over-Expression Promoted Cytokines Release by MSCs

**Fig. 4:**
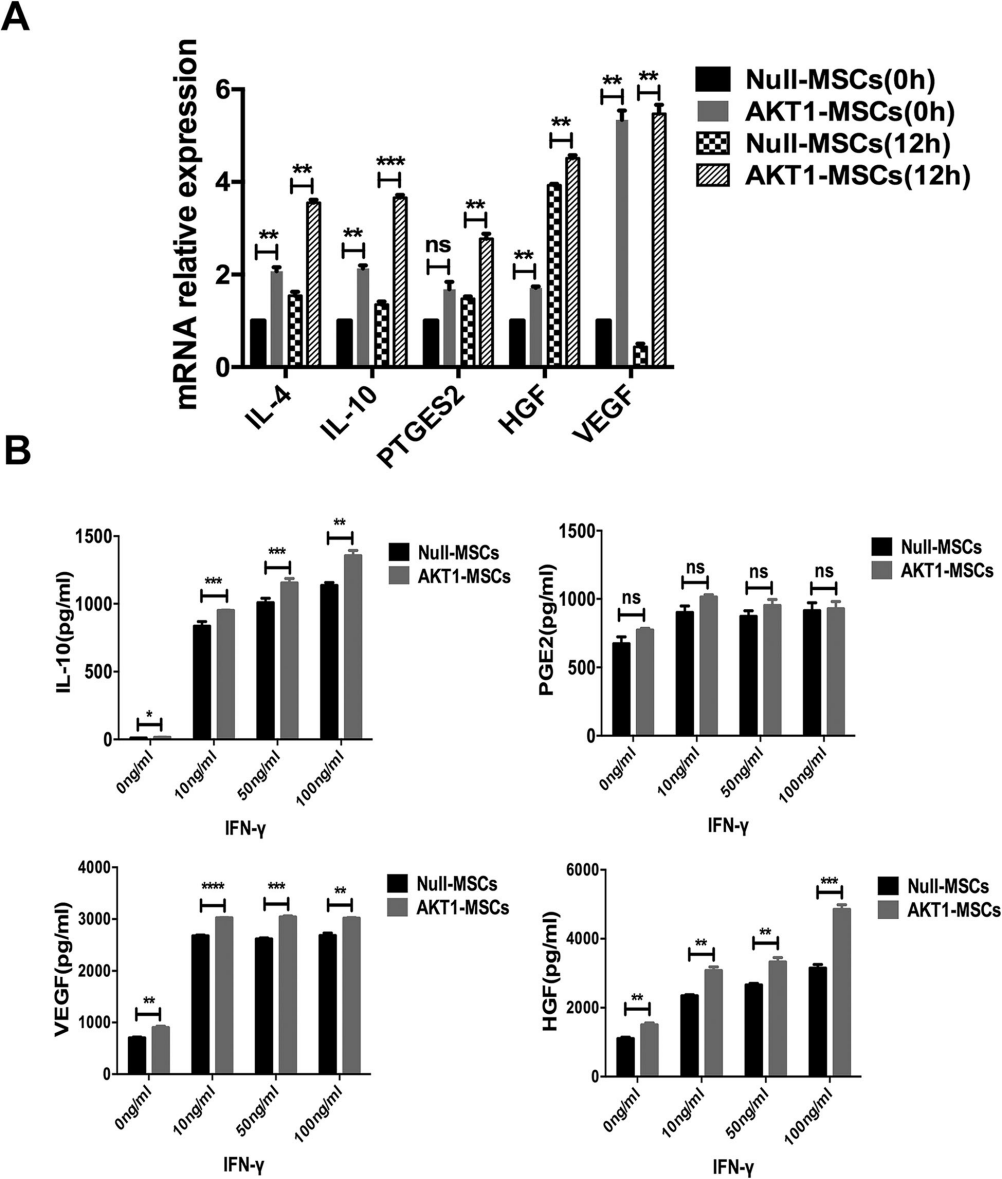
AKT1 over-expression promoted cytokines release by MSCs. **a** The mRNA expression of IL-4, IL-10, PTGES2, HGF and VEGF in Null-MSCs and AKT1-MSCs were measured by qRT-PCR under routine culture condition and IFN-γ stimulation condition. The gene transcript levels were normalized to those of GAPDH and set to 1 in Null-MSCs untreated group. **b** ELISA analysis for detecting IL-10, PGE2, VEGF and HGF protein expression in MSCs culture supernatant after IFN-γ stimulation for 24h. The data are presented as the means ± SD of three independent experiments. *P < 0.05, **P < 0.01, ***P < 0.001, ****P < 0.0001

It has been reported that MSCs has an immunosuppressive capacity, which is partially mediated by its secreted soluble factors, such as cytokines, chemokines and growth factors [27]. We investigated whether overexpression of AKT1 enhanced immunosuppressive cytokines release by MSCs under inflammatory environment. Null-MSCs and AKT1-MSCs were treated with IFN-γ (100ng/ml) for 12h and then collected. qRT-PCR was performed to examine the mRNA expression of immunosuppressive cytokines interleukin-4 (IL-4), interleukin-10 (IL-10) and prostaglandin E synthase 2 (PTGES2). As shown in Fig. 4a, the mRNA of IL-4, IL-10 and PTGES2 were significantly upregulated in AKT1-MSCs compared to Null-MSCs. Interestingly, we also found the upregulation of hepatocyte growth factor (HGF) and vascular endothelial growth factor (VEGF) in AKT1-MSCs compared to Null-MSCs. These results were further confirmed at protein level by measuring cytokines in cell culture supernatant with ELISA. AKT1-MSCs had a higher level of IL-10, HGF and VEGF after simulation with different dosages of IFN-γ for 24h (Fig. 4b). The above results suggested that AKT1-MSCs could not only release more anti-inflammatory cytokines (IL-10) but also more growth factors (HGF, VEGF) under inflammatory environment, which contributed to its protective effect in immune-mediated hepatitis.

### AKT1-MSCs Was Superior to Null-MSCs in Ameliorating ConA-Induced Liver Injury

**Fig. 5:**
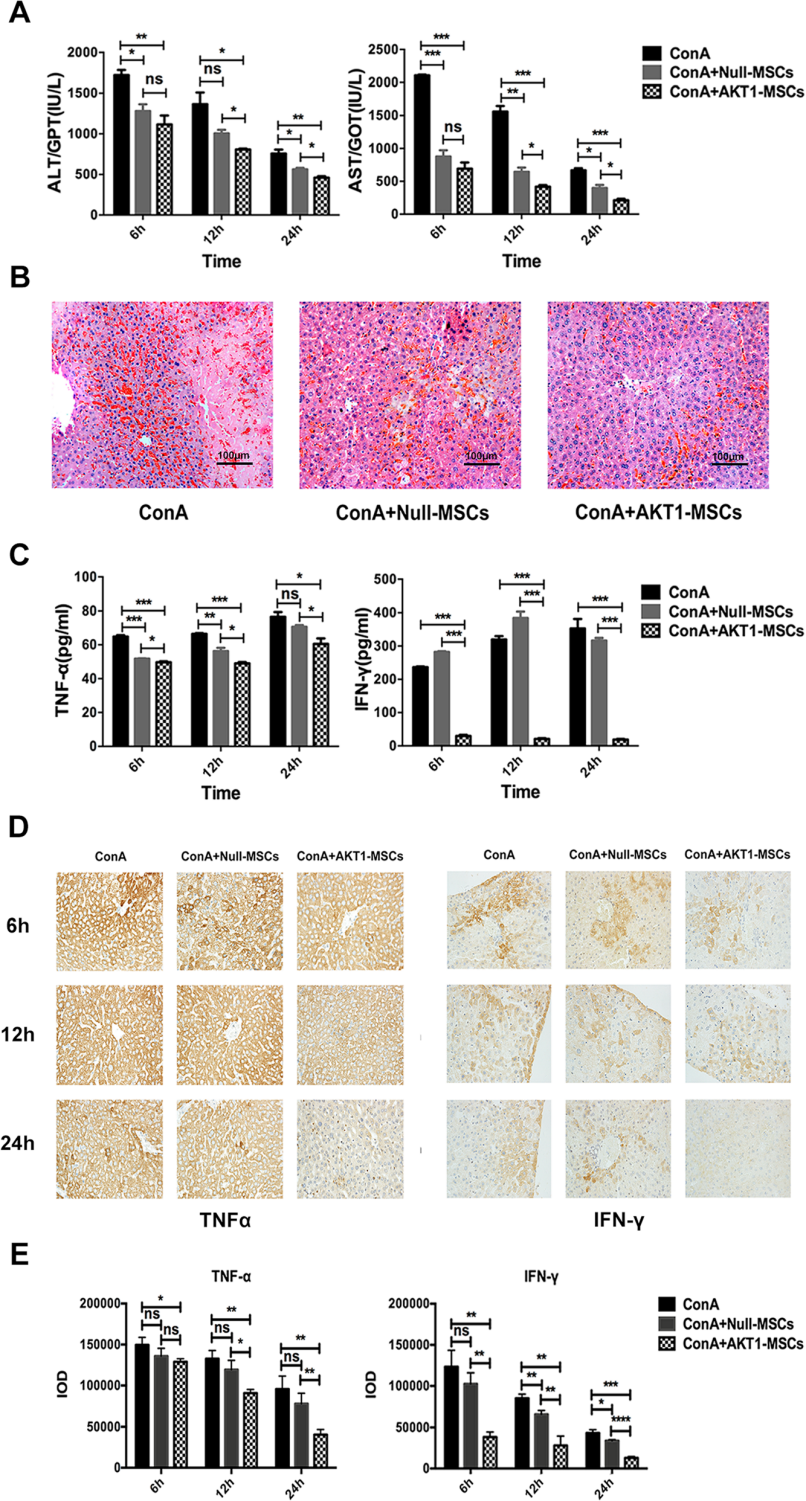
AKT1-MSCs ameliorated ConA-induced liver injury. Female C57BL/6 mice were intravenously injected with PBS (negative control), Null-MSCs (5×10^6^), or AKT1-MSCs (5×10^6^) 30min after ConA (20mg/kg) administration. **a** Serum levels of ALT and AST were measured at 6h, 12h, and 24h after MSCs injection. **b** Representative photographs of H&E-stained liver tissue in each group were shown. Scale bar = 100μm. **c** The expression of TNF-α and INF-γ in serum was measured by ELISA at 6h, 12h and 24h after MSCs injection. **d** Intrahepatic TNF- α and INF-γ protein levels were measured by immunohistochemical staining at 6h, 12h and 24h after MSCs injection. **e** The expression quantifications of TNF- α and INF-γ were analyzed by Image-Pro Plus 6.0. IOD (Integrated Option Density) was checked out by calculating the area and the density of the brown color in IHC staining. The data are presented as the means ± SD of three independent experiments. *P < 0.05, **P < 0.01, ***P < 0.001, ****P < 0.0001

To investigate the role of MSCs in liver aGVHD, we use a ConA-induced liver injury model to imitate liver aGVHD as they both closely related to T cell activation and pro-inflammatory cytokines release in terms of hepatotoxic mechanism. ConA (20mg/kg) was injected intravenously into mice to induce liver injury and 5×10^6^ Null-MSCs or AKT1-MSCs was injected into recipient mice 30 min after ConA administration. We examined the influences of Null-MSCs and AKT1-MSCs on liver injury by evaluating serum transaminase levels and liver tissue sections hematoxylin-eosin (H&E). Serum transaminase assay revealed a significant decrease in alanine aminotransferase (ALT) and aspartate transaminase (AST) after MSCs injection, and there was more in mice received AKT1-MSCs (Fig. 5a). The protective effect of AKT1-MSCs was further confirmed by histopathology, which showed that hepatocellular injuries in AKT1-MSCs recipients were less severe than those in Null-MSCs recipients, as demonstrated by a significant reduction in the severity of tissue necrosis (Fig. 5b).

To further elucidate the molecular mechanism underlying the protective effect of MSCs on ConA-induced liver injury, cytokine expression was measured in serum and liver tissue at various time points after injection of Null-MSCs and AKT1-MSCs. As shown in Fig. 5c, administration of MSCs significantly reduced serum level of TNF-α and there was an additional and significant reduction of TNF-α in AKT1-MSCs recipients compared to Null-MSCs recipients. Null-MSCs did not reduce serum level of IFN-γ, whereas AKT1-MSCs was associated with a significant reduction of IFN-γ (Fig. 5c). Furthermore, MSCs decreased ConA-induced pro-inflammatory cytokine gene expression in liver tissues as demonstrated by immunohistochemical staining of TNF-α and INF-γ. There was an intense staining pattern of TNF-α and INF-γ in ConA-treated mice without MSCs administration. However, in MSCs injection groups, TNF-α and INF-γ staining levels were much lower and the expressions were even lower in AKT1-MSCs injected mice compared with Null-MSCs injected mice (Fig. 5d, e). Since ConA is known to cause acute liver failure via an immunologic activated cytokine response syndrome with TNF-α and INF-γ as the pivotal mediators, these data implied that AKT1-MSCs had a superior protective effect on immune-mediated hepatitis.

## Discussion

MSCs derived from bone marrow may include HSCs and multipotent progenitor cells. In this study, we performed phenotypic analysis of the BM-MSCs by flow cytometry to determine its type and purity. As shown in Fig. 1b, these cells barely expressed hematopoietic cell surface markers such as CD34 (HSCs), CD45 (leukocytes), CD11b (monocytes/macrophages) and CD3 (lymphocytes), but they expressed MSCs specific markers CD29 and CD44, which confirmed its identity.

BM-MSCs has benefited a significant number of patients suffering from aGVHD. However, the therapeutic effects are not always reliably achieved [6–10]. Exploring new ways to improve its therapeutic efficiency would provide great clinical benefits. In this study, we genetically modified mouse BM-MSCs with AKT1 gene and evaluated its function. We used a ConA-induced liver injury model to mimic liver aGVHD as they are similar in terms of the hepatotoxic mechanism. ConA-induced hepatitis depends on the activation of T cells by macrophages in the presence of ConA, followed by the release of a variety of cytokines such as TNF-α and IFN-γ to mediate inflammatory liver damage [28–30]. Liver aGVHD is caused by mature donor T cells that recognize alloantigens presented by host antigen-presenting cells (APCs), followed by a rapid burst of T-lymphocyte proliferation and tissue damage induced by activated T lymphocytes, cytokines and cells of the innate immune system [4]. They are both T-cell dependent liver injury disease. IFN-γ, a potent pro-inflammatory cytokine produced by activated T-cells, NK cells, NK/T cells and macrophages, is considered as a major pathogenic factor related to both ConA-induced liver injury and liver aGVHD [4, 22]. Thus, we compared cell apoptosis and the paracrine function of Null-MSCs and AKT1-MSCs under IFN-γ stimulation condition. As shown in Fig. 3a, b, cell apoptosis was significantly reduced in AKT1-MSCs group as compared to Null-MSCs group. AKT1-MSCs also produced more immunosuppressive factors (e.g. IL-10) and growth factors (e.g., HGF and VEGF) critical for MSC-mediated tissue repair (Fig. 4a, b).

*In vivo* studies showed that both Null-MSCs and AKT1-MSCs homed to the damaged liver section in ConA-administrated mice, but not to the normal liver section in PBS-administrated mice, suggesting that the injected MSCs preferably migrated to injured tissue (Fig. 3c). This is consistent with a previous study showing that MSCs preferentially homed to the site of myocardial ischemia but not to normal uninjured myocardium [20]. Interestingly, we also found that AKT1-MSCs has a homing advantage over Null-MSCs, as there was more AKT1-MSCs than Null-MSCs in liver section on day 1 after MSCs administration (Fig. 3c). It has been reported that several adhesion molecules and chemokines were involved in the homing process of MSCs, such as chemokine receptor type 4 (CXCR4), vascular cell adhesion molecule 1 (VCAM-1) and integrin β1 [14, 31]. Further experiments need to be done to elucidate the mechanism of the improved homing ability of AKT1-MSCs. The homed MSCs survived longer in AKT1-MSCs group than in Null-MSCs group, which is in keeping with *in vitro* study showing AKT1 over-expression endowed BM-MSCs with a survival advantage.

The liver function of the AKT1-MSCs administered mice was significantly improved compared to the Null-MSCs administered mice. This conclusion was based on the measurements of serum ALT and AST (Fig. 5a) and histological staining of liver tissue sections (Fig. 5b). More importantly, the expression of IFN-γ, which plays a pivotal role in liver inflammatory reaction, was also significantly reduced in AKT1-MSCs group, suggesting AKT1-MSCs was more effective than Null-MSCs in rescuing liver injury (Fig. 5c–e).

## Conclusions

In summary, the results presented here showed that AKT1-modified BM-MSCs had a survival advantage and an enhanced immunomodulatory function compared to unmodified BM-MSCs, leading to better amelioration of ConA-induced liver injury. We further demonstrated that AKT1-modified BM-MSCs exerted a superior therapeutic effect through paracrine action by secreting more immunosuppressive factors and growth factors. The genetically modified MSCs with AKT1 could be used to prevent and ameliorate liver aGVHD after hematopoietic stem cell transplantation and other immunity-associated liver injuries. Clinical studies are needed to assess its therapeutic potentials.

## Methods

### MSCs Culture and Identification

Mouse (C57/B6) bone marrow MSCs was obtained from the Cyaen company (Guangzhou, China). MSCs was cultured in Dulbecco's Modified Eagle Medium/Nutrient Mixture F-12 (DMEM/F-12, GibcoTM, USA) supplemented with 10% MSC-qualified Fetal Bovine Serum (GibcoTM, USA), 1% penicillin/streptomycin (GibcoTM, USA), and 1% GlutaMAX™-1 Supplement (GibcoTM, USA). At 80-90% confluency, cells were detached by trypsin/EDTA and passaged at a split ratio of 1:3. After detachment, cells were collected and incubated with the following phycoerythrin-conjugated antibodies: CD29, CD44, Sca-1, CD11b, CD3, CD45, CD34, MHC II and KLF1. The percentage of fluorescence-highlighted cells was determined by flow cytometry (Canto II, BD, USA).

### Generation of AKT1 Over-Expressing MSCs

pMSCV-MCS-IRES-eGFP and pMSCV-AKT1-IRES-eGFP, provided by Victor J Dzau’s laboratory (Department of Medicine, Duke University School of Medicine), were co-transfected with packaging vectors VSVG and pKAT into HEK293T cells using lipofectamine 2000 (Invitrogen, USA) to produce retroviruses [20]. At 48h and 72h after transfection, viral supernatants were collected and concentrated to 100-fold by ultracentrifugation after pelleting cell debris. AKT1 and control viruses were transduced into MSCs following a modified protocol reported by Rick et al [32]. In brief, immediately after detachment by trypsin, MSCs at passage 3 was mixed with virus at a multiplicity of infection (MOI) of 40 for 16h in 0.5 ml complete medium in the presence of 8 μg/ml polybrene (Sigma, USA), followed by replacement with fresh complete medium. This procedure was repeated twice and the cells were expanded thereafter. GFP expression was visualized with a Zeiss Axio A1 fluorescence microscope. Successfully transduced MSCs was sorted by BD FACS Aria III System for GFP+ cells and expanded for another two passages before being used for the experiments.

### RNA Isolation and Real-Time Quantitative PCR (qRT-PCR)

RNA was extracted with TRIzol reagent (Invitrogen, USA) and reverse-transcribed into cDNA using M-MLV Reverse Transcriptase (Life Technologies, USA). qRT-PCR was performed on a 7500 Real-Time PCR system (Applied Biosystems, USA) with SYBR Green PCR kit (Takara, Japan) following the manufacturer’s instructions. Primers used for PCR were listed as follows: (1) AKT1 5'-AACGGACTTCGGGCTGTG-3' and 5'-TTGTCCTCCAGCACCTCAGG-3'; (2) HGF 5'-ATCCACGATGTTCATGAGAG-3' and 5'-GCTGACTGCATTTCTCATTC-3'; (3) VEGF 5'-GCGGGCTGCCTCGCAGTC-3' and 5'-TCACCGCCTTGGCTTGTCAC-3'; (4) IL-4 5'-AGAGATCATCGGCATTTTGAACGA-3' and 5'- CGAGCTCACTCTCTGTGGTGT TCT-3'; (5) IL-10 5'-CTTGCACTACCAAAGCCACA-3' and 5'-GTTATTGTCTTCCCGGC TGT-3'; (6) PTGES2 5'-ACTTCCACTCCCTGCCCT-3' and 5'-CAGGTACCAAGGCTGGAT GT-3', respectively.

### Western Blot Assay

Western blot assay was performed as previously described [33] with primary antibodies targeting AKT (Cell Signaling Technology, USA) and p-AKT (Cell Signaling Technology, USA). β-actin (Abcam, USA) was used as internal control.

### Apoptosis Assessment

5×10^5^ cells were collected and stained with Annexin V-Alexa Fluor 647-A and PI (BioLegend, USA) according to the manufacturer’s instructions. Apoptosis assay was performed with flow cytometry (LSRII, BD, USA) and Annexin V-positive cells were identified as apoptotic cells.

### ConA-Induced Liver Injury Mouse Model and MSCs Transplantation

To establish ConA-induced liver injury mouse model, 30 female BALB/c (H2d) mice (6~8 week, weighing 18-20 g) were randomly divided into three groups and 20mg/kg ConA was injected intravenously. At 30 minutes after ConA administration, mice in group A (n = 10) were injected with PBS via the tail vein. Group B (n = 10) received 5 × 10^6^ Null-MSCs and Group C (n = 10) received 5 × 10^6^ AKT1-MSCs via the tail vein. The extent of liver injury was evaluated by serum transaminase assay and histopathological examination of liver tissue samples taken from sacrificed mice. All animal experiments were approved by the Institutional Animal Care and Use Committee of Peking Union Medical College.

### Histopathological Analysis, Immunohistochemistry and Serum Transaminase Assay

At the time of sacrifice, liver tissue samples were collected, fixed in 4% formaldehyde for 24h and embedded in paraffin. Then 3-μm liver sections were deparaffinized and fixed. Section samples were stained with hematoxylin solution (Sigma-Aldrich, Germany) for 5 min followed by eosin for 5 min. TNF-α and IFN-γ in liver sections were detected by immunohistochemistry (BOSTER.Bio, China) according to the manufacturer’s instructions. For serum transaminase assay, serum ALT and AST levels were measured with an automated biochemical analyzer (Roche Integra 800, USA).

### Enzyme-Linked Immunosorbent Assay

TNF-α, INF-γ, IL-10, HGF, VEGF and PGE2 in serum and MSCs culture medium were measured using enzyme-linked immunosorbent assay (ELISA) kits (Liankebio, China) according to the manufacturer’s instructions.

### In Vivo Fluorescent Imaging

Null-MSCs and AKT1-MSCs were intravenously injected into the tail veins of BALB/c mice 30 min after ConA administration. Twenty-four hours after ConA injection, mice were euthanized and the livers were extracted. Tracking of *in vivo* transplanted MSCs localized to liver was performed using an IVIS fluorescence imaging system (Xenogen IVIS-200 Optical in Vivo Imaging System, Caliper Life Sciences Inc, Hopkinton, MA).

### Statistical Analysis

Results were calculated as means ± SD. Statistical significance was evaluated with an unpaired Student’s t-test and *P* < 0.05 was considered statistically significant.

## Data Availability

Datasets and materials are available by the corresponding author.

## Abbreviations

aGVHD: Acute graft-versus-host disease
Allo-HSCT: Allogeneic hematopoietic stem cell transplantation
ALT: Alanine aminotransferase
APCs: Antigen-presenting cells
AST: Aspartate transaminase
BM-MSCs: Bone marrow-derived mesenchymal stem cells
ConA: Concanavalin A
CXCR4: Chemokine receptor type 4
H&E: Hematoxylin-eosin
HGF: Hepatocyte growth factor
IFN-γ: Interferon-γ
IL-10: Interleukin-10
IL-4: Interleukin-4
KLF1: Kruppel-like factor1
MHC II: Major histocompatibility complex II
mRNA: Messenger ribonucleic acid
MSCs: Mesenchymal stem cells
PGE-2: Prostaglandin E2
PTGES2: Prostaglandin E synthase 2
qRT-PCR: Real-time polymerase chain reaction
TNF-α: Tumor necrosis factor α
VCAM-1: Vascular cell adhesion molecule 1
VEGF: Vascular endothelial growth factor
